# Effect of social support on depression of internet addicts and the mediating role of loneliness

**DOI:** 10.1186/1752-4458-8-34

**Published:** 2014-08-16

**Authors:** Fei He, Qin Zhou, Jing Li, Rong Cao, Hao Guan

**Affiliations:** 1 School of Public Management, Northwest University, Xi’an, China; 2 Department of Burns and Cutaneous Surgery, Xijing Hospital, Xi’an, China

**Keywords:** Internet addiction, Depression, Social support, Loneliness, Structural equation modeling

## Abstract

**Background:**

Many studies have determined the existence of an extremely close association between Internet addiction and depression. However, the reasons for the depression of Internet addicts have not been fully investigated.

**Aim:**

This cross-sectional study aims to explore the factors that influence depression among Internet addicts.

**Methods:**

A total of 162 male Internet addicts completed the Emotional and Social Loneliness Scale, Multidimensional Scale of Perceived Social Support, and Self-Rating Depression Scale.

**Results:**

Loneliness and lack of social support are significantly correlated with depression among Internet addicts. Structural Equation Modeling results indicate that social support partially mediates loneliness and depression.

**Conclusions:**

Both social support and loneliness were negatively associated with depression of Internet addicts whereas loneliness plays a mediating role between social support and depression.

## Background

Since the late 1990s, the Internet has entered a rapid development track in China [1]. On one hand, the Internet has allowed adolescents to enjoy the unprecedented convenience and pleasure brought about by modern technological civilization in the virtual world of the Internet, particularly in learning and communicating [2]. On the other hand, the Internet is a "double-edged sword" because of its negative effects on the multifaceted psychological development of the adolescents. Most interesting of these negative effect is Internet addiction [3-5]. Internet addiction, also known as pathological Internet use (PIU), is a multi-dimensional syndrome comprising cognitive and behavioral symptoms, which generally leads to social withdrawal, truancy, loneliness, and depression [6-11].

Studies have found a very close association between Internet addiction and depression [12-14]. Young reported that level of depression is highly correlated with Internet addiction [15]. Other researchers found that Internet use and addiction increase the risk of depression among adults and is related with depression and suicidal ideation among adolescents [16,17]. The depression scores of Internet addicts were found to be significantly higher than those of non-addicts [18]. Researchers believe that this depression is due to the addiction of adolescents to the virtual world, which impedes their relationships in the real world. In effect, they lack face-to-face communication and real social support, which increases their susceptibility to depression [19,20].

Loneliness is a painful subjective experience or psychological feeling, which is characterized by the lack of satisfactory relationships [21]. Sullivan believes that loneliness is an unpleasant experience arising from unfulfilled intimate or close interpersonal needs [22]. Michela et al. [23] view loneliness as the unpleasant experience of individuals brought about by the realization that a gap exists between the expected and actual results in relation to the social network model. Russell, Peplau, and Ferguson believe that loneliness is a subjective social isolation state stemming from a person’s painful experience of not being accepted by others [24]. The relationship among social support, depression, and loneliness among adolescents has been defined by various studies. Many studies have focused on the effects of social support from a specific source, and showed a significant negative correlation between the support from parents, companions, or teachers and the depression and loneliness of adolescents [19,25-28].

Previous findings have revealed that Internet addicts are prone to depression, and depression is associated with loneliness and the lack of social support. However, these findings failed to combine the two issues: (1) Can social support and loneliness affect depression of Internet addicts? (2) What is the trilateral relationship among social support, loneliness and depression? We hypothesized that both social support and loneliness have significant impacts on the depression of Internet addicts, and that loneliness plays a mediating role between social support and depression.

## Methods

### Participants

The participants consisted of 1,024 male undergraduate students, with ages between 19 and 23, from four Xi’an universities, which have comprehensive course offerings. A total of 1,024 questionnaires were distributed, and 1,023 were collected. Thirty-three questionnaires were partially filled out and thus, were discarded. A total of 990 questionnaires were considered valid. The Internet addiction test employed the clinical diagnosis questionnaire designed by Young. The revised Internet addiction test comprises eight items answerable by yes or no. The subject was regarded as an Internet addict if five or more of his answers were positive [29]. Of the 990 subjects, 162 (16.36%) were Internet addicts. Subsequently, these participants were asked to take more measures.

### Instruments

#### Multidimensional scale of perceived social support

The Multidimensional Scale of Perceived Social Support is a 12-item instrument with a seven-point scale (1 = strongly disagree; 7 = strongly agree) that measures three sources of support, namely, family, friends, and significant others [30]. Items in this scale include "My family really tries to help me", "My friends really try to help me", and "There is a special person in my life who cares about my feelings". The scale was translated into Chinese by Chou [31], and has been proved to have good validity and reliability in Chinese populations. In the present study, the Cronbach alpha coefficient for Multidimensional Scale of Perceived Social Support was 0.808.

#### Social and emotional loneliness scale

The Social and Emotional Loneliness Scale was developed by Wittenberg, which consists of 10 items designed to assess social loneliness (SL) and emotional loneliness (EL). Responses to each item are given on a five-point Likert-type scale ranging from 1 (never) to 5 (very often). It includes items such as, "1 have a really nice set of friends"; "I have friends and acquaintances with who I like to be together". Scale scores are the sum of items with reverse coding of relevant items [32]. The Social and Emotional Loneliness Scale was translated into Chinese by Kong and You [33]. In the current study, the Cronbach alpha coefficient for the Social and Emotional Loneliness Scale was 0.787.

#### Self-rating depression scale (SDS)

Self-rating Depression Scale (SDS), developed by Zung, is a self-report measure of depression consisting of 20 items, with a four-point scale ranging from a little of the time (1) to most of the time (4). Of the 20 items, 10 are worded positively and 10 are worded negatively. The former 10 items are reversed items. The validity and the reliability of the SDS have been reported [34]. The researcher translated the 20-item version of the Self-rating Depression Scale into Chinese, and the Chinese vision of the scale has been proved to have good validity and reliability [35]. In the current study, the Cronbach alpha coefficient for SDS was 0.851.

### Data analysis

Firstly, the intercorrelations of all the latent variables were calculated to test the significant of correlation coefficient. Then, a two-step procedure introduced by Anderson and Gerbing was adapted to analyses the mediation effect in order to confirm the structural relations of the latent structured model [36]. Firstly, the measurement model of the four latent variables was tested to assess the extent of goodness of fit represented by its indicators respectively [37]. If index of confirmatory measurement model meet the requirements, then the maximum likelihood estimation would be used to test the SEM [38]. All the above analysis was conducted in AMOS 17.0 program.

The following four indices were utilized to evaluate the goodness of fit of the model: (a) Chi-square statistic (χ^2^), χ^2^/df, (b) the Standardized Root Mean Square Residual (SRMR), (c) the Root Mean Square Error of Approximation (RMSEA), and (d) the Comparative Fit Index (CFI) [39-41]. In this study, a model was considered to have a good fit if all the path coefficients were significant at the level of 0.05, χ^2^/df was below 5, SRMR was below 0.08, RMSEA was below 0.08, and CFI was 0.95 or more.

## Results

### Descriptive statistics and correlation analysis

Means, standard deviations, and intercorrelations of all variables are presented in Table 1. The results indicate that social support is negatively correlated with loneliness (r = -0.44, p < 0.01) and depression (r = -0.42, p < 0.01). By contrast, loneliness is positively correlated with depression (r = 0.33, p < 0.01).

**Table 1:** Means, standard deviations, and correlations of the variables of interest

	**Mean**	**SD**	**1**	**2**	**3**
1. Social support	66.81	12.17	1		
2. Loneliness	32.15	6.42	-0.44^**^	1	
3. Depression	66.03	8.08	-0.42^**^	0.33^**^	1

Note: ^**^p < 0.01.

### Measurement model

Confirmatory factor analysis (CFA) was adopted to assess whether or not the measurement model adequately fits the sample data. The initial test of the measurement model revealed a satisfactory fit [χ^2^ (df = 17) = 21.50, χ^2^/df = 1.27; RMSEA = 0.041; SRMR = 0.046 and CFI = 0.993]. All the factor loadings for the indicators on the latent variables were significant (p < 0.001), indicating that all the latent constructs are well-represented by their indicators.

### Structural model

**Figure 1:**
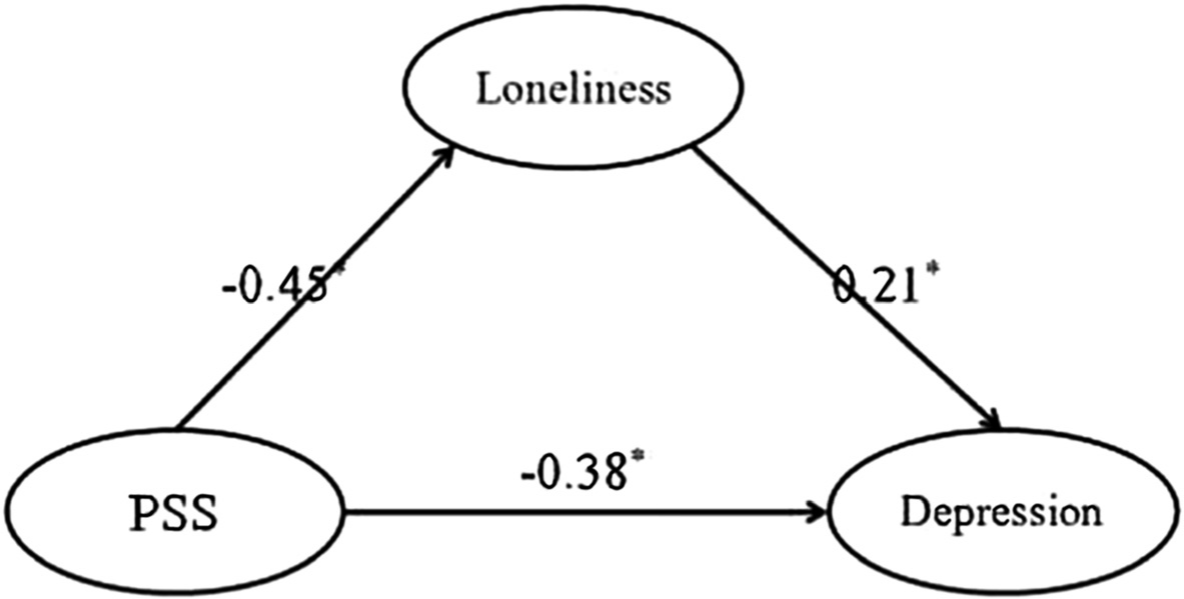
**The impact of social support and loneliness on depression of Internet addicts.** Note: Factor loadings are standardized (p < 0.05).

To analyze the mediation effect, structural equation modeling (SEM) was used. First, the direct effect of social support on depression without mediators was tested. The directly standardized path (β = -0.47, p < 0.001) was significant. After which, a partially-mediated model containing a mediator (loneliness) and a direct path from social support to depression was tested. All the path coefficients were statistically significant, see Figure 1: The results showed an acceptable fit of the model to the data [χ^2^ (df = 17) = 21.50, χ^2^/df = 1.27; RMSEA = 0.041; SRMR = 0.046 and CFI = 0.993]. These results showed that social support and loneliness have significant effects on depression among Internet addicts.

### Confidence interval of direct and indirect effects

The mediating effects of loneliness on social support and depression were tested for significance by adopting the Bootstrap estimation procedure in AMOS (a bootstrap sample of 1,000 was specified). Sobel test, the commonly employed method for examining statistical significance of a mediation effect, which involves computing the ratio of products of direct effects to their estimated standard error, was not considered. The test requires the products of direct effects to follow a normal distribution, which does not generally occur, resulting in the reduction of statistical efficacy. By contrast, the bootstrap test relies on 95% confidence intervals from the empirical distribution of indirect effect estimates. According to Mackinnon [40,42,43], the bootstrap method yields the most accurate confidence intervals for indirect effects. Table 2 shows the indirect effects and their associated 95% confidence intervals. The indirect effect of social support on depression through loneliness is significant.

**Table 2:** Direct and indirect effects and 95% confidence intervals for the final model

**Model pathways**	**Estimated effect**	**95% CI**	**95% CI**
		**Lower bonds**	**Up bonds**
Direct effect			
Social support → Depression	-0.378	-0.192	-0.551
Social support → Loneliness	-0.455	-0.209	-0.659
Loneliness → Depression	0.211	0.035	0.430
Indirect effect			
Social support → Loneliness → Depression	-0.096	-0.012	-0.246

## Discussion

The current study examines the effect of loneliness and social support on depression among Internet addicts. As expected, the correlational findings of the study indicate that social support has a negative relationship with depression and loneliness. The result of SEM indicates that loneliness partially mediates the relationship between social support and depression.

Furthermore, social support has a significant predication effect on depression, which is consistent with the previous conclusion that states that social support is a kind of available resource, which allows Internet addicts to re-evaluate the produced negative emotions that directly reduce their depression and anxiety to some extent [44]. In addition, a good social support can reduce the feeling of pressure, which motivates an individual to improve one’s perceived self-response capacity toward alleviating the evaluation of the severity of stressful events, and reducing the adverse effects of the stressful experience [45]. Social support is significantly associated with loneliness: the lesser the feeling of social support, the higher the feeling of loneliness [46,47]. Loneliness is essentially a state where an individual feels the lack of satisfactory relationships and a subjective psychological feeling or experience where a gap arises between the personal desire for interaction and the actual level of interaction [48]. The current study found that loneliness can partially mediate the effect of social support on the depression of Internet addicts. Loneliness is a closed and diffused mental state, and a depressed mood arising from the feeling of isolation and exclusion; and the individual’s perception of the quality and quantity of his own social interaction, often accompanied by loneliness and helplessness, as well as other negative emotions and spiritual emptiness, which consequently produces depression [21,49].

The study concludes that the Internet causes addiction, which in turn leads to the lack of social support. The lack of social support, on one hand, can directly lead to depression; on the other hand, it can indirectly affect depression through the mediation of loneliness. In summary, this study has provided insights into the relationship among social support, loneliness, and depression. In particular, social support acts as a protective factor by decreasing the feelings of loneliness, which in turn creates a beneficial effect on depression. Thus, to enhance the mental health level of Internet addicts, adequate social support should be provided to address the feelings of isolation and exclusion. Despite these achievements, the present study has limitations. First, the study’s correlational cross-sectional nature prohibits the drawing of any causal relationships among the variables. Thus, caution must be exercised when interpreting the results of meditational analyses on cross-sectional data. Future longitudinal or experimental studies should be conducted to facilitate more causal evaluations. Second, loneliness was the only potential mediator that was examined despite the presence of other mediators that capable of playing an important role in the relationship between social support and depression. Thus, other variables, such as self esteem, should be examined. Third, the data in this study were collected only through self-report scales. The use of multiple methods for evaluation may have decreased the "subjectivity" limitation of the findings.

## Competing interests

The authors declare that they have no competing interests.

## Authors’ contributions

Conceived and designed the experiments: FH, HG. Performed the experiments: FH, QZ, JL, RC, HG. Analyzed the data: JZ, JP. Contributed reagents/materials/analysis tools: FH, HG. Wrote the paper: FH, HG. All authors read and approved the final manuscript.
